# Complete mitochondrial genome of the hawkmoth *Notonagemia analis scribae* (Lepidoptera: Sphingidae)

**DOI:** 10.1080/23802359.2016.1176883

**Published:** 2016-06-20

**Authors:** Min Jee Kim, Jong Seok Kim, Iksoo Kim

**Affiliations:** Department of Applied Biology, College of Agriculture & Life Sciences, Chonnam National University, Gwangju, Republic of Korea

**Keywords:** Bombycoidea, mitochondrial genome, *Notonagemia analis scribae*, Sphingidae

## Abstract

Currently, the mitochondrial genome of only two species of Sphingidae have been completely sequenced. For the phylogenetic study of Bombycoidea (including Bombycidae, Saturniidae and Sphingidae) using mitochondrial genomes (mitogenomes), more species are required as a basis for future research. In the present study, we sequenced the complete mitogenome of the hawkmoth, *Notonagemia analis scribae* (Lepidoptera: Sphingidae), to enrich the Sphingidae database. The length of the *N. a. scribae* genome was 15,303 bp with a typical set of genes (13 protein-coding genes [PCGs], 2 rRNA genes, and 22 tRNA genes), and one major non-coding A + T-rich region. The *COI* gene had a CGA start codon, which is the start codon for this gene in the majority of lepidopteran species, whereas other PCGs began with ATN codons. A 318-bp A + T-rich region harbored the blocks of conserved sequences that are typically found in lepidopteran insects, excluding a poly-A stretch, which is typically found at the end of the A + T-rich region. Phylogenetic analysis using the 13 PCGs indicated that *N. a. scribae* grouped together with two within-familial species, *Sphinx morio* and *Manduca sexta*, with the highest nodal support both by Bayesian inference and maximum-likelihood methods, forming the Sphingidae monophyletic group.

The lepidopteran family Sphingidae (superfamily Bombycoidea) of hawk moths include ∼1450 species of relatively large-sized moths (van Nieukerken et al. 2011). Previously, phylogenetic relationships of three families of Bombycoidea (Bombycidae, Saturniidae and Sphingidae) were studied (Zwick et al. 2011), but no mitogenome-based study has been carried out due to limited taxon diversity in the Sphingidae: only *Sphinx morio* and *Manduca sexta* are available (Cameron & Whiting 2008; Kim et al. 2013). Thus, more species from a diverse taxonomic group are necessary for future mitogenome-based phylogenetic studies. Therefore, in the present study, the complete mitogenome of the hawkmoth *Notonagemia analis scribae* was sequenced, annotated and used for preliminary phylogenetic analysis using the available species of Bombycoidea.

One adult was captured at Mt. Jiri, Jeollabuk-do Province, South Korea (35°20′01′′ N, 127°36′59′′ E). A voucher specimen was deposited at Chonnam National University, Gwangju, Korea. Total DNA was used as the template to amplify three long overlapping fragments (*COI*-*ND4*, *ND5*-*lrRNA* and *lrRNA*-*COI*). Subsequently, 26 short overlapping fragments were amplified using the long fragments as templates. All primers used were Lepidoptera-specific primers designed previously (Kim et al. 2012).

The complete mitogenome of *N. a. scribae* (GenBank accession number KU934302) was 15,303 bp and consisted of two rRNAs, 22 tRNAs, 13 protein-coding genes (PCGs), and 1 major non-coding region (the A + T-rich region). Regarding genome size, *N. a. scribae* falls between two available sphingids (15,299 bp in *S. morio* and 15,516 bp in *M. sexta*) and the gene arrangement is identical to that of other ditrysian Lepidoptera, including Sphingidae, that have the order *trnM-trnI-trnQ* (where the underline indicates a gene inversion) between the A + T-rich region and *ND2* (Cameron & Whiting 2008; Kim et al. 2011, 2013). Twelve of the 13 PCGs started with ATN codons (data not shown), but the *COI* gene began with CGA (arginine). The 318-bp A + T-rich region displayed several Lepidoptera-specific features, such as the ATAGA motif and adjacent poly-T stretch, and microsatellite-A/T repeat, but a poly-A stretch, which is typically found at the end of the A + T-rich region (upstream of 5′-end of *trnM*) was found as a mixture of A and T nucleotides (AATATAAATATTA), similar to other sequenced sphingids (data not shown).

Phylogenetic analysis using nucleotide sequences of 13 PCGs was performed on 12 species in three families of Bombycoidea, including *N. a. scribae*. Bayesian inference (BI) and maximum-likelihood (ML) methods were performed using the GTR + GAMMA + I model in CIPRES Portal v. 3.1 (Miller et al. 2010). The two approaches generated an identical topology, but nodal support varied (Figure 1). *N. a. scribae*, grouped together with two within-familial species, *S. morio* and *M. sexta*, with the highest nodal support (BI, 1.0; ML, 100%), forming the Sphingidae monophyletic group. Furthermore, each Bombycidae and Saturniidae also formed monophyletic groups with the highest nodal support in both analyses. Although there have been several hypotheses regarding the phylogenetic relationships among bombycoid families (Zwick et al. 2011), our limited data supported the sister relationship between Bombycidae and Sphingidae, with the placement of Saturniidae as the basal lineage of the two families. For robust inference of bombycoid families, an increased taxon diversity is required.

**Figure 1.**
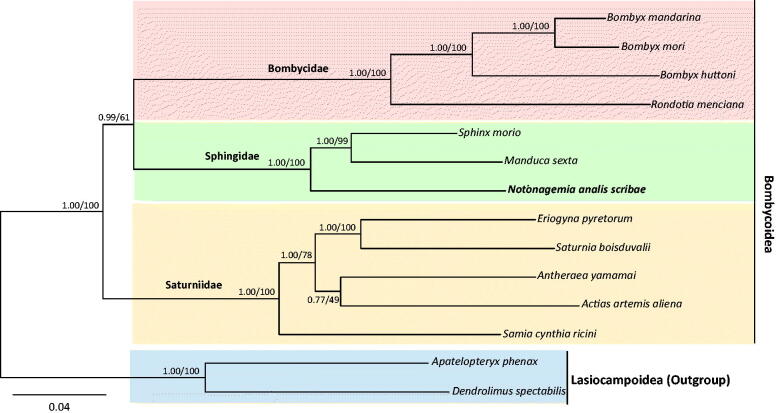
Phylogenetic tree for Bombycoidea. Bayesian inference (BI) and maximum-likelihood (ML) methods produced the same topology based on concatenated 13 PCGs. The numbers at each node specify Bayesian posterior probabilities (%) by BI analysis (first value) and bootstrap percentages of 1000 pseudoreplicates by ML analysis (second value). The scale bar indicates the number of substitutions per site. Two species of Lasiocampoidea were utilized as outgroups. GenBank accession numbers are as follows: *Notonagemia analis scribae*, KU934302 (the present study), *Sphinx morio*, KC470083 (Kim et al. 2013); *Manduca sexta,* EU286785 (Cameron & Whiting 2008); *Bombyx mandarina*, AB070263 (Yukuhiro et al. 2002); *Rondotia menciana,* KJ647172 (Kim et al. 2016)*; Bombyx huttoni*, KP216766 (Peng et al. 2015); *Bombyx mori*, AF149768 (Unpublished); *Actias artemis aliena*, KF927042 (Park et al. 2016); *Samia cynthia ricini*, JN215366 (Kim et al. 2012); *Saturnia boisduvalii*, EF622227 (Hong et al. 2008); *Antheraea yamamai*, EU726630 (Kim et al. 2009); *Eriogyna pyretorum*, FJ685653 (Jiang et al. 2009); *Dendrolimus spectabilis*, KM244678 (Tang et al. 2014); and *Apatelopteryx phenax*, KJ508055 (Timmermans et al. 2014).
